# Supplementary material for: Anatomy and mechanics of tsetse fly blood feeding
Source: eLife. 2026 Oct 13;15:RP110151. doi: 10.7554/eLife.110151 (PMC13661044; doi:10.7554/eLife.110151)
Supplement: Supplementary file 1 [file elife-110151-mdarchecklist1.docx]

**Materials Design Analysis Reporting (MDAR)**

**Checklist for Authors**

The [MDAR framework](https://osf.io/xfpn4/) establishes a minimum set of requirements in transparent reporting mainly applicable to studies in the life sciences.

*eLife* asks authors to **provide detailed information within their article** to facilitate the interpretation and replication of their work. Authors can also upload supporting materials to comply with relevant reporting guidelines for health-related research (see [EQUATOR Network](http://www.equator-network.org/%20)), life science research (see the [BioSharing Information Resource](http://biosharing.org/)), or animal research (see the [ARRIVE Guidelines](http://www.plosbiology.org/article/info:doi/10.1371/journal.pbio.1000412) and the [STRANGE Framework](https://doi.org/10.1038/d41586-020-01751-5); for details, see *eLife*’s [Journal Policies](https://reviewer.elifesciences.org/author-guide/journal-policies)). Where applicable, authors should refer to any relevant reporting standards materials in this form.

For all that apply, please note **where in the article** the information is provided. Please note that we also collect information about data availability and ethics in the submission form.

**Materials:**

| **Newly created materials** | **Indicate where provided: section/figure legend** | **N/A** |
| --- | --- | --- |
| The manuscript includes a dedicated "materials availability statement" providing transparent disclosure about availability of newly created materials including details on how materials can be accessed and describing any restrictions on access. | Methods describe the custom measurement apparatus and skin-equivalent preparation. No dedicated statement on access to newly created apparatus or materials is provided. |  |

| **Antibodies** | **Indicate where provided: section/figure legend** | **N/A** |
| --- | --- | --- |
| For commercial reagents, provide supplier name, catalogue number and [RRID](https://scicrunch.org/resources), if available. | Not applicable; no antibody-based assays are described. |  |

| **DNA and RNA sequences** | **Indicate where provided: section/figure legend** | **N/A** |
| --- | --- | --- |
| Short novel DNA or RNA including primers, probes: Sequences should be included or deposited in a public repository. | Not applicable; no novel DNA or RNA sequences are reported. |  |

| **Cell materials** | **Indicate where provided: section/figure legend** | **N/A** |
| --- | --- | --- |
| Cell lines: Provide species information, strain. Provide accession number in repository OR supplier name, catalog number, clone number, OR RRID. | Methods, Fly infection: Trypanosoma brucei brucei EATRO 1125, serodeme AnTat 1.1; culture and infection protocols are cited. No established human cell line is described. |  |
| Primary cultures: Provide species, strain, sex of origin, genetic modification status. | Methods, Origin of probing substrates and Ethical clearance statement: primary human epidermal keratinocytes and dermal fibroblasts from male donors aged 2–5 years. Genetic modification status is not stated. |  |

| **Experimental animals** | **Indicate where provided: section/figure legend** | **N/A** |
| --- | --- | --- |
| Laboratory animals or Model organisms: Provide species, strain, sex, age, genetic modification status. Provide accession number in repository OR supplier name, catalog number, clone number, OR RRID. | Methods, Fly keeping and force measurements; Figures 2 and 8. The Discussion identifies G. morsitans for blood-meal measurements. Sex and quantitative sample numbers are reported; colony/strain, source, age and genetic modification status are not explicitly specified in the methods. |  |
| Animal observed in or captured from the field: Provide species, sex, and age where possible. | Methods, Origin of probing substrates: young roe deer (Capreolus capreolus), cow skin from a farmer, and Nile monitor (Varanus niloticus), museum specimen ZMFK 84206. Sex and full age information are not reported for all specimens. |  |

| **Plants and microbes** | **Indicate where provided: section/figure legend** | **N/A** |
| --- | --- | --- |
| Plants: provide species and strain, ecotype and cultivar where relevant, unique accession number if available, and source (including location for collected wild specimens). | Not applicable; no plant material is described. |  |
| Microbes: provide species and strain, unique accession number if available, and source. | Methods, Fly infection: T. brucei brucei EATRO 1125, serodeme AnTat 1.1; published culture/infection protocol cited. |  |

| **Human research participants** | **Indicate where provided: section/figure legend) or state if these demographics were not collected** | **N/A** |
| --- | --- | --- |
| If collected and within the bounds of privacy constraints report on age, sex, gender and ethnicity for all study participants. | Methods, Origin of probing substrates and Ethical clearance statement: commercial abdominal skin from a 36-year-old female, Fitzpatrick type 2; primary cells from male donors aged 2–5 years. Other donor demographics are not reported. |  |

**Design:**

| **Study protocol** | **Indicate where provided: section/figure legend** | **N/A** |
| --- | --- | --- |
| If the study protocol has been pre-registered, provide DOI. For clinical trials, provide the trial registration number OR cite DOI. | No preregistration or clinical trial registration is reported; this is not described as a clinical trial. |  |

| **Laboratory protocol** | **Indicate where provided: section/figure legend** | **N/A** |
| --- | --- | --- |
| Provide DOI OR other citation details if detailed step-by-step protocols are available. | Methods cite protocols for fly infection, CLSM, fixation, freeze substitution, friction-force measurement and skin-equivalent culture. The Methods also describe acquisition and image-processing settings. |  |

| **Experimental study design (statistics details) *** | | |
| --- | --- | --- |
| **For in vivo studies: State whether and how the following have been done** | **Indicate where provided: section/figure legend. If it could have been done, but was not, write “not done”** | **N/A** |
| Sample size determination | Sample numbers are reported in Figure 2, Figure 8, Figure 8—figure supplement 2 and Methods. A prospective power calculation or rationale for sample-size determination is not reported. |  |
| Randomisation | Randomisation procedures are not reported in the manuscript; whether randomisation was performed cannot be established from the available records. |  |
| Blinding | Blinding procedures are not reported in the manuscript; whether blinding was performed cannot be established from the available records. |  |
| Inclusion/exclusion criteria | Prospectively defined inclusion/exclusion criteria are not reported. The Dryad KNOWN_LIMITATIONS.md documents an unresolved omitted attachment trial in a stored summary; the original record is retained. |  |

| **Sample definition and in-laboratory replication** | **Indicate where provided: section/figure legend** | **N/A** |
| --- | --- | --- |
| State number of times the experiment was replicated in the laboratory. | Figure 2: 10 flies per sex; repeated measurements averaged per fly. Figure 8 and Methods: 20–25 flies per substrate. Figure 8—figure supplement 2: 24 feeding events. Independent repetition counts for each imaging procedure are not reported. |  |
| Define whether data describe technical or biological replicates. | Figure 2 distinguishes individual flies from repeated within-fly trials and plots per-fly means. Figure 8 reports probing observations per substrate. Raw recording blocks, derived exports and repeated summaries are not independent biological replicates; see Dryad documentation. |  |

| **Ethics** | **Indicate where provided: section/submission form** | **N/A** |
| --- | --- | --- |
| Studies involving human participants: State details of authority granting ethics approval (IRB or equivalent committee(s), provide reference number for approval. | Methods, Ethical clearance statement: University of Würzburg ethics approvals 182/10 and 280/18-SC; informed consent from the legal representatives of donors is reported. |  |
| Studies involving experimental animals: State details of authority granting ethics approval (IRB or equivalent committee(s), provide reference number for approval. | Methods describe tsetse experiments and use of vertebrate tissue. No specific animal ethics approval or explicit exemption statement is reported. |  |
| Studies involving specimen and field samples: State if relevant permits obtained, provide details of authority approving study; if none were required, explain why. | Methods, Origin of probing substrates, identifies commercial, agricultural, hunter-supplied and museum sources. Permit or exemption details for specimen acquisition are not reported. |  |

| **Dual Use Research of Concern (DURC)** | **Indicate where provided: section/submission form** | **N/A** |
| --- | --- | --- |
| If study is subject to dual use research of concern regulations, state the authority granting approval and reference number for the regulatory approval. | No DURC designation or regulatory approval is reported in the manuscript. |  |

**Analysis:**

| **Attrition** | **Indicate where provided: section/figure legend** | **N/A** |
| --- | --- | --- |
| Describe whether exclusion criteria were pre-established. Report if sample or data points were omitted from analysis. If yes, report if this was due to attrition or intentional exclusion and provide justification. | Prospective exclusion criteria are not reported. Dryad KNOWN_LIMITATIONS.md records discrepancies between source observations and stored summaries, including an omitted attachment trial of unknown historical rationale. Original data remain unchanged. |  |

| **Statistics** | **Indicate where provided: section/figure legend** | **N/A** |
| --- | --- | --- |
| Describe statistical tests used and justify choice of tests. | Methods, Friction force measurement, Proboscis force measurement and Statistical analysis; legends for Figures 2 and 8 and Figure 8—figure supplement 1: Friedman, Mann–Whitney, Kruskal–Wallis and one-sample Wilcoxon tests. A complete justification of test selection and all multiple-comparison procedures is not reported. |  |

| **Data availability** | **Indicate where provided: section/submission form** | **N/A** |
| --- | --- | --- |
| For newly created and reused datasets, the manuscript includes a data availability statement that provides details for access (or notes restrictions on access). | Data availability section; Supplementary Files 1–3 and Videos 1–4; Dryad DOI 10.5061/dryad.n02v6wxdh. The deposit is in curation and covers quantitative/force data. Complete microscopy stacks and anatomical reconstruction files are not included. |  |
| When newly created datasets are publicly available, provide accession number in repository OR DOI and licensing details where available. | Dryad: https://doi.org/10.5061/dryad.n02v6wxdh; CC0. Public release is pending completion of curation. |  |
| If reused data is publicly available provide accession number in repository OR DOI, OR URL, OR citation. | No external dataset reused for quantitative analysis is identified; prior literature is cited in the manuscript. |  |

| **Code availability** | **Indicate where provided: section/figure legend** | **N/A** |
| --- | --- | --- |
| For any computer code/software/mathematical algorithms essential for replicating the main findings of the study, whether newly generated or re-used, the manuscript includes a data availability statement that provides details for access or notes restrictions. | Methods identify image-processing software, acquisition software and statistical packages, and describe the force equations. Dryad provides source workbook formulas, open-format exports and a Python ACQ-reading example. |  |
| Where newly generated code is publicly available, provide accession number in repository, OR DOI OR URL and licensing details where available. State any restrictions on code availability or accessibility. | Dryad DOI 10.5061/dryad.n02v6wxdh includes additional_force_data/examples/read_acq_example.py for accessing the deposited recordings. This is an access example, not a complete reproduction of all analyses. |  |
| If reused code is publicly available provide accession number in repository OR DOI OR URL, OR citation. | Methods cite Fiji/ImageJ and identify commercial packages. The Dryad documentation links bioread: https://github.com/uwmadison-chm/bioread. External software retains its own licence. |  |

**Reporting:**

The MDAR framework recommends adoption of discipline-specific guidelines, established and endorsed through community initiatives.

| **Adherence to community standards** | **Indicate where provided: section/figure legend** | **N/A** |
| --- | --- | --- |
| State if relevant guidelines (e.g., ICMJE, MIBBI, ARRIVE, STRANGE) have been followed, and whether a checklist (e.g., CONSORT, PRISMA, ARRIVE) is provided with the manuscript. | This MDAR checklist is supplied. Adherence to other named community reporting guidelines is not stated in the manuscript. |  |

* We provide the following guidance regarding transparent reporting and statistics; we also refer authors to [Ten common statistical mistakes to watch out for when writing or reviewing a manuscript](https://doi.org/10.7554/eLife.48175).

**Sample-size estimation**

- You should state whether an appropriate sample size was computed when the study was being designed
- You should state the statistical method of sample size computation and any required assumptions
- If no explicit power analysis was used, you should describe how you decided what sample (replicate) size (number) to use

**Replicates**

- You should report how often each experiment was performed
- You should include a definition of biological versus technical replication
- The data obtained should be provided and sufficient information should be provided to indicate the number of independent biological and/or technical replicates
- If you encountered any outliers, you should describe how these were handled
- Criteria for exclusion/inclusion of data should be clearly stated
- High-throughput sequence data should be uploaded before submission, with a private link for reviewers provided (these are available from both GEO and ArrayExpress)

**Statistical reporting**

- Statistical analysis methods should be described and justified
- Raw data should be presented in figures whenever informative to do so (typically when N per group is less than 10)
- For each experiment, you should identify the statistical tests used, exact values of N, definitions of center, methods of multiple test correction, and dispersion and precision measures (e.g., mean, median, SD, SEM, confidence intervals; and, for the major substantive results, a measure of effect size (e.g., Pearson's r, Cohen's d)
- Report exact p-values wherever possible alongside the summary statistics and 95% confidence intervals. These should be reported for all key questions and not only when the p-value is less than 0.05.

**Group allocation**

- Indicate how samples were allocated into experimental groups (in the case of clinical studies, please specify allocation to treatment method); if randomization was used, please also state if restricted randomization was applied
- Indicate if masking was used during group allocation, data collection and/or data analysis
