# Supplementary material for: Anatomy and mechanics of tsetse fly blood feeding
Source: eLife. 2026 Oct 13;15:RP110151. doi: 10.7554/eLife.110151 (PMC13661044; doi:10.7554/eLife.110151)
Supplement: Peer review history

# Editor report: eLife Assessment

**DOI:** 10.7554/eLife.110151.3.sa0

**Authors:** Gordon J Berman

**Affiliations:** Emory University United States

**Keywords:** Compelling, Fundamental

This work provides a **fundamental** advance through a detailed, integrative analysis of how the tsetse fly feeds on blood, demonstrating that successful penetration depends on subtle structural adaptations rather than extreme forces or unusual anatomy. By combining high-resolution imaging, innovative biomechanical measurements, and experiments on artificial skin, the study offers complementary and **compelling** evidence, with clear data supporting a robust mechanistic interpretation. These findings have broad significance, as they clarify the biomechanics of vector feeding and have implications for the transmission of diseases such as African trypanosomiasis across diverse hosts.

# Referee report: Reviewer #3 (Public review)

**DOI:** 10.7554/eLife.110151.3.sa1

Summary:

Human and animal trypanosomiasis are fatal illnesses caused by African trypanosomes transmitted by tsetse flies during a bloodmeal. Thus, tsetse fly feeding is the key physical step in disease transmission to mammals. Tsetse fly feeding is not a new story, but it is revisited here through the application of sophisticated imaging techniques and novel biomechanical methods of analysis. The author's aim is to provide a high-resolution picture of the structures and forces involved in feeding to provide mechanistic insights into the process of feeding, from attachment, penetration, drinking and retraction of the feeding parts.

Largely the authors have achieved their aims. They (i) examine the structures and forces involved in attachment; (ii) they provide detailed multi image analysis of the proboscis providing insights into its probing ability and physical mechanism of penetration; (iii) they conduct a controlled analysis of the physical forces involved in penetration and report that they are in the low nM range, not especially strong but much higher that the mosquito bite and finally they provide a first analysis of blood uptake during feeding.

Strengths:

The study images the tsetse fly feeding structures in unprecedented detail, with resolution to the uM scale, in 3-D, and during feeding. The resulting images are dramatic and insightful (and beautiful and frightening!) that researchers interested in trypanosomes, tsetse flies or blood feeding by flies in general will want to see.

They conclude that flies attach strongly to smooth surfaces, because of interactions possible via the array of acanthae of the pulvillus pad at the ends of the tarsi. The estimated attachment forces are similar in male & female flies, in the low mM range (they look impressively strong in video 1). They provide a very striking analysis of the proboscis and labellum and associated tooth structures (Figs 4 & 5). I recall many years ago observing that tsetse flies are messy feeders, and these structures, especially the rasping teeth structures on the reverse folded labial tips explain why! This seems more like a chainsaw than a jigsaw in action, but the authors are probably correct that these structures and probing/retraction mechanism explain many features of tsetse fly feeding and their ability to feed on a wide range of hosts with very different skin types.

The impressive aspect of this paper is the range of imaging techniques, (CLSM, SEM, uCT, FIB SEM), the quality of the images which attests to the obvious care taken with sample preparation. The biomechanically analysis, especially the penetration analysis is impressive. Finally, the paper is clearly written and presented, it was a very easy read and overall, a very engaging study.

Weaknesses:

I suppose it could be said that the paper is a descriptive study; it doesn't really test a hypothesis but that is not a prerequisite for publication. Perhaps the least convincing prats are the imaging of the flexible v rigid parts of the structures, which is based on amount of resilin (flexible) and chitin-protein (stiff) based on their autofluorescence. In seems odd that the joints would be less blue (stiffer) in Fig 1i, or what the blue structures correspond to in Fig. 6B-D.

Comments on revised version.

In revised version these issues have been satisfactorily addressed

# Author comment: Author response

**DOI:** 10.7554/eLife.110151.3.sa2

**Authors:** Löwe Stephan, Laura Hauf, Elisabeth Meyer-Natus, Dianah Katiti, Dennis Petersen, Alexander Kovalev, Sebastian Büsse, Anna Steyer, Yoko Matsumura, Wolfgang Böhme, Daniel Masiga, Stanislav Gorb, Markus Engstler

**Affiliations:** University of Würzburg Würzburg Germany; University of Würzburg Würzburg Germany; University of Würzburg Würzburg Germany; International Centre of Insect Physiology and Ecology Nairobi Kenya; Kiel University Kiel Germany; Kiel University Kiel Germany; Department of Functional Morphology and Biomechanics, Institute of Zoology, Kiel University Kiel Germany; Max Planck Institute for Multidisciplinary Sciences Göttingen Germany; Kiel University Kiel Germany; Zoological Research Museum Alexander Koenig Bonn Germany; International Centre of Insect Physiology and Ecology Nairobi Kenya; University of Kiel University Germany; University of Würzburg Würzburg Germany

The following is the authors’ response to the original reviews.

**Public Reviews:**

**Reviewer #1 (Public review):**

Summary:

This manuscript provides a comprehensive and mechanistic analysis of how tsetse flies feed on blood across a wide range of host skin types. The authors combine detailed anatomical characterization of the feeding apparatus with quantitative measurements of mechanical properties, probing forces, and blood uptake, complemented by experiments using artificial skin. They show that tsetse flies do not rely on extreme forces or uniquely specialized structures, but instead on subtle and highly efficient structural and mechanical adaptations (such as the toothed labellum and coordinated proboscis movements) to achieve effective blood pool feeding. The study successfully moves beyond descriptive anatomy to a quantitative, functional analysis that explains how feeding is accomplished across diverse substrates.

Strengths:

A major strength of the work is the impressive integration of multiple complementary approaches. Advanced imaging tools provide a convincing three-dimensional view of the proboscis, labellum, and associated structures, while direct force measurements and blood intake quantification place these observations on a solid quantitative footing. The use of artificial skin with different mechanical properties is particularly powerful, as it allows structure-function relationships to be tested under controlled and reproducible conditions. Together, these datasets provide strong and coherent support for the authors' central conclusions. The quantitative treatment of feeding mechanics represents a significant advance over largely descriptive prior work by others (e.g., Gibson W et al 2017) and establishes a valuable mechanistic insight for studying blood feeding in insect vectors more broadly.

Weaknesses:

The study focuses almost entirely on uninfected flies and does not address how infection might alter feeding mechanics or performance. Previous work has shown that trypanosome infection can affect salivary gland function and feeding time (Van Den Abbeele et al 2010), and even cause damage to mouthparts, all of which can influence feeding behavior and efficiency. While this does not detract from the technical quality or the core findings of the study, a more explicit discussion of these biological variables would help place the results in a broader transmissionrelevant context and clarify how generalizable the conclusions are to natural infection settings.

We thank the reviewer for this important comment. While our study focused on uninfected flies, we agree that parasite infection may influence feeding performance and should therefore be considered when assessing the broader relevance of our findings. Previous studies have shown that trypanosome infections can alter salivary gland physiology and saliva composition (Van Den Abbeele et al., 2010; Matetovici et al., 2016). In addition, transcriptomic analyses suggest that infection with *T. congolense* may affect the molecular and physiological state of the proboscis (Awuoche et al., 2017). However, there is currently no direct evidence that these changes translate into fundamental alterations of the mechanical properties or function of the mouthparts themselves, which were the primary focus of our study. We have now expanded our manuscript to discuss this (lines 508-520):

"It is also important to note that our experiments were conducted using uninfected flies. Previous studies have shown that trypanosome infection can alter feeding behaviour, leading to increased probing activity and prolonged feeding times (Jenni et al., 1980; Van den Abbeele et al., 2010). These effects have primarily been attributed to infection-induced changes in saliva composition and the resulting interactions with host blood (Van den Abbeele et al., 2010). However, a different study found no significant effects of infection with either salivary gland-resident *T. brucei* or with proboscis-colonizing species such as *T. congolense* and *T. vivax* on Glossina feeding behaviour (Moloo, 1983). Furthermore, although infection-associated transcriptional changes in the salivary glands and proboscis have been reported (Awuoche et al., 2017; Matetovici et al., 2016), there is currently no direct evidence that trypanosome infection alters the mechanical properties or function of the mouthparts themselves."

Overall, this is an outstanding and carefully executed study that will have a significant impact on the fields of vector biology and parasite transmission.

**Reviewer #2 (Public review):**

Summary:

This manuscript presents an impressively detailed, multidisciplinary analysis of the mechanics of blood feeding in Glossina spp. Combining SEM, CLSM, µCT, FIB-SEM, macro-videography, and quantitative force measurements, the authors characterize the structures and biomechanics of attachment, proboscis deployment, tissue penetration, and blood uptake. They also examine interactions with diverse host-type substrates, from human skin equivalents to cow, deer, and lizard skin, and integrate these with force measurements to quantify penetration and retraction dynamics.

The work's key conclusion is that the tsetse fly does not rely on any single exceptional morphological innovation, but rather uses a suite of subtle structural features and retractive forces to feed efficiently across diverse hosts. This result is novel, insightful, and evolutionarily compelling. Overall, this is a strong manuscript that combines methodological sophistication with biological relevance. It should be of high interest to researchers studying vector biology, biomechanics, parasite transmission, and vector-host interactions.

Strengths:

(1) The combination of SEM, CLSM, µCT, and FIB-SEM provides an unusually comprehensive anatomical characterization of the tsetse feeding apparatus.

(2) The direct measurement of proboscis penetration and retraction forces across diverse substrates is highly original and fills a major knowledge gap in vector-host interaction mechanics.

(3) The study bridges morphology, mechanics, behavior, and host tissue properties, which strengthens the overall conclusions.

(4) Imaging of trypanosomes within the hypopharynx and surrounding tissue during feeding provides new information about parasite delivery mechanisms.

Main Comments:

(1) The authors conclude that feeding versatility arises from the sum of subtle adaptations. This interpretation is reasonable, but it would help to sharpen which findings most robustly support this statement. For example, the relative similarity of proboscis forces across skin types is compelling evidence that the proboscis is broadly tuned rather than specialized. The observation that tsetse targets softer interscale regions on lizard skin suggests behavioural selectivity, not morphological specialisation. It would strengthen the discussion to highlight which data most directly refute the hypothesis of a unique specialization.

We thank the reviewer for this comment. To address this point more explicitly and to sharpen the interpretation of our findings, we have expanded the final conclusion in the Discussion (lines 528544):

"Ultimately, the objective of this study was to investigate how tsetse flies can feed on a seemingly random selection of animals with highly diverse skin structures. In our detailed anatomical studies and force measurements, we did not identify a single dominant trait that explains the fly's feeding versatility.

Instead, our results indicate that this capability emerges from the combined effect of multiple, more subtle traits. In particular, the proboscis generates broadly similar penetration forces across a wide range of skin types, suggesting a generalised mechanical mechanism rather than hostspecific optimisation. The intricate architecture of the labellum and the strong retractile forces during probing likely contribute to efficient penetration and blood pool formation across heterogeneous substrates. Behaviourally, tsetse flies further increase feeding success by flexibly targeting mechanically favourable sites, such as the softer interscale regions on lizard skin, rather than relying on specialised morphological adaptations.

This composite strategy likely reflects evolutionary fine-tuning that enables the broad host range of tsetse flies. By allowing efficient blood feeding across diverse vertebrate hosts, this versatility may also have facilitated the ecological success and transmission opportunities of African trypanosomes.”

(2) A central finding is that retraction forces exceed penetration forces across substrates, implying that backward pulling is a key component of wound creation. However, the biological interpretation could be deepened. Specifically, do the authors believe retraction serves primarily to enlarge the pool-feeding site? How does this compare mechanically to mosquito fascicle oscillation or other blood-feeding arthropods (especially other flies such as those in the tabanidae family)? Could retraction forces contribute to anchoring or resisting host grooming behaviors?

The stronger retraction forces observed during probing indeed suggest that backward pulling is not a passive withdrawal, but likely an active component of tissue disruption. As discussed in the manuscript (lines 475–482), we interpret these repeated pullback movements, together with the outward-facing prestomal teeth of the everted labellum, primarily as a mechanism to enlarge the feeding lesion and improve access to blood, consistent with the blood pool feeding strategy of tsetse flies. To make this more clear, we have added a half sentence to line 482 "..., thereby creating a larger blood pool for feeding."

We also already compare this mechanism to mosquito feeding mechanics in the discussion (starting from line 487). In mosquitoes, high-frequency fascicle oscillations are thought to reduce insertion resistance and facilitate minimally invasive capillary feeding. Although we also observed oscillatory movements during tsetse feeding (Video 4), the underlying mechanical strategy appears fundamentally different. In contrast to the mosquito’s system optimized for delicate penetration, the tsetse proboscis appears adapted for forceful tissue disruption during pool feeding. Notably, the oscillations observed in tsetse flies seem to occur during active blood uptake rather than initial tissue penetration. Consequently, the functional role of these oscillations in tsetse flies remains unclear. We have now addressed this more specifically in the discussion (lines 490-495):

"Oscillatory movements were also observed during tsetse probing (Video 4). Notably, these oscillations appeared predominantly during active blood uptake rather than during the initial penetration phase, suggesting that they are associated with ingestion rather than insertion. Whether they facilitate blood flow, prevent occlusion of the feeding canal, or simply reflect pump activity remains unknown."

When looking at other species, stable flies (*Stomoxys*) may represent a particularly relevant comparison because they employ a similar penetration mechanism and are pool feeders with prominent prestomal teeth (Krenn and Aspöck. Function and evolution of the mouthparts of blood-feeding Arthropoda. Arthropod structure and development, 2012). In contrast, tabanids employ a different mouthpart architecture with rasping/cutting structures but without comparable prestomal teeth. Whereas mosquito mouthparts have been described as functioning like a syringe, and we compare the tsetse proboscis to a saw, tabanid mouthparts have been likened to scissors (Krenn and Aspöck. Function and evolution of the mouthparts of blood-feeding Arthropoda. Arthropod structure and development, 2012). Although tabanids are also known to inflict substantial tissue damage, it remains unclear whether their feeding movements produce retraction-dominated force patterns comparable to those we observed in tsetse flies.

Lastly, we agree that the elevated resistance generated during retraction may contribute to withstanding host defensive behaviour such as shake-off responses. Structurally, the orientation of the prestomal teeth and the architecture of the everted labellum could provide temporary anchoring during feeding, as we have already briefly discussed in the manuscript (lines 458– 461). However, while stronger anchoring may increase feeding stability, it could also increase the risk of injury to the fly if detected by the host. Compared to other pool-feeding flies such as stable flies, tsetse flies have been reported to respond more readily to host defensive behaviour (Schofield and Torr. A comparison of the feeding behaviour of tsetse and stable flies. Medical and Veterinary Entomology, 2002). We therefore currently consider anchoring to be a possible secondary function but lack direct experimental evidence to assess its practical importance.

(3) The study analyzes a diverse set of substrates, which is a strength. However, some caveats deserve explicit discussion. Human skin equivalents and dermal equivalents lack the full mechanical complexity of real skin (e.g., innervation, perfusion, tension). Frozen or ethanol-stored samples, particularly reptile skin, may also exhibit altered mechanical properties compared to live tissues. These limitations do not undermine the findings but should be explicitly acknowledged as they influence the interpretation of absolute force magnitudes.

The reviewer raises a valid point regarding the interpretation of absolute force magnitudes across the measured substrates. We have therefore added a clarifying statement to the discussion (lines 469-476):

"When interpreting absolute force magnitudes, it is important to bear in mind that our samples do not fully recapitulate physiological conditions. Skin explants and skin equivalents may behave differently to skin under active perfusion and native tissue tension, as may our fixed and frozen animal skin samples. Nevertheless, comparative force measurements revealed consistent biomechanical signatures across substrates, suggesting that the observed force patterns reflect fundamental aspects of the feeding mechanism that are likely relevant in vivo.”

(4) The SEM and FIB-SEM images showing trypanosomes in the hypopharynx and surrounding tissue during penetration are visually striking and suggest rapid dispersal. It would be helpful to connect these observations more clearly to the kinetics of parasite deposition and whether mechanical tissue laceration is likely to increase inoculation efficiency. Without conducting additional experiments, the authors could discuss whether these findings support or modify existing models of salivary-gland-derived parasite release.

We have now expanded the Discussion to clarify that our observations of trypanosomes in the hypopharynx are consistent with the established model of salivary-gland-derived parasite release during probing and feeding, in which infective metacyclic trypanosomes are delivered with saliva into the host tissue. Furthermore, the presence of trypanosomes beyond the immediate feeding canal supports rapid parasite dispersal following inoculation, as described in previous work (Reuter et al., 2023). In this context, the tissue laceration generated by the tsetse proboscis may facilitate local parasite distribution by creating a larger, mechanically disrupted feeding lesion. However, our data provide high-resolution structural snapshots and were not designed to quantify deposition kinetics or inoculation efficiency. We therefore refrain from concluding that mechanical laceration increases transmission efficiency and instead view this as a plausible consequence that should be tested directly in future work. Specifically, we have added this paragraph to the discussion (521-527):

"Overall, our observations of trypanosomes within the fly's hypopharynx, labial gutter, and host tissue are consistent with the established model of salivary-gland-derived parasite release during probing and feeding. Their presence beyond the immediate feeding canal is consistent with rapid local dispersal following inoculation, as described previously (Reuter et al., 2023). This process may be facilitated by the extensive tissue disruption caused by the tsetse mouthparts, although this hypothesis will require direct experimental testing."

(5) The authors demonstrate that tsetse attachment abilities fall within the range of generalist insects and are far lower than those of obligate ectoparasites. However, the manuscript could discuss how attachment forces relate to the tsetse's ecological context, e.g., whether their attachment is generally brief, whether host shaking strongly selects for grip strength, etc. Is there evidence that other Glossina species or tabanids with different host preferences show variation in attachment performance? This would broaden the relevance of the findings.

Tsetse flies are obligate blood feeders, but host contact is typically brief and frequently interrupted by host defensive behaviour. As a result, selection may favour rapid and efficient feeding rather than exceptionally strong attachment. This interpretation is supported by Schofield and Torr (A comparison of the feeding behaviour of tsetse and stable flies. Medical and Veterinary Entomology, 2002), showing that tsetse flies experience more feeding interruptions than the stable fly *Stomoxys calcitrans*, despite completing successful blood meals in less time. These differences are consistent with life-history theory (Anderson and Roitberg. Modelling trade-offs between mortality and fitness associated with persistent blood feeding by mosquitoes. Ecology Letters, 1999), which predicts that long-lived species with low reproductive rates, such as tsetse flies, should be less willing to risk injury by persisting on a host than shorter-lived, more fecund species. Against this background, our finding that tsetse attachment forces fall within the range reported for generalist insects, appears biologically plausible. Their attachment performance needs to be functionally sufficient for brief feeding events rather than maximized for prolonged host retention.

We are not aware of comparative biomechanical data on attachment performance across different *Glossina* species or tabanids. We agree that such comparative studies would be valuable to test whether differences in host preference and feeding ecology correlate with variation in attachment capacity.

(6) In video 4, could the authors clarify whether the observed maxillary vibrations are hypothesized to reduce penetration resistance or serve another function?

The vibrations of the maxilla specifically appear during active blood uptake rather than during initial tissue penetration, suggesting they are linked to the ingestion phase. Whether they serve a mechanical function, such as facilitating blood flow or preventing canal occlusion, or represent a passive consequence of pump activity, remains unclear. We consider this an open and interesting question that warrants dedicated investigation.

We have therefore clarified that the functional significance of these oscillations remains unresolved to date (lines 490-495). This reads: “Oscillatory movements were also observed during tsetse probing (Video 4). Notably, these oscillations appeared predominantly during active blood uptake rather than during the initial penetration phase, suggesting that they are associated with ingestion rather than insertion. Whether they facilitate blood flow, prevent occlusion of the feeding canal, or simply reflect pump activity remains unknown.”

**Reviewer #3 (Public review):**

Summary:

Human and animal trypanosomiasis are fatal illnesses caused by African trypanosomes transmitted by tsetse flies during a bloodmeal. Thus, tsetse fly feeding is the key physical step in disease transmission to mammals. Tsetse fly feeding is not a new story, but it is revisited here through the application of sophisticated imaging techniques and novel biomechanical methods of analysis. The authors aim to provide a high-resolution picture of the structures and forces involved in feeding to provide mechanistic insights into the process of feeding, from attachment, penetration, drinking and retraction of the feeding parts.

Largely, the authors have achieved their aims. They (i) examine the structures and forces involved in attachment; (ii) they provide detailed multi image analysis of the proboscis providing insights into its probing ability and physical mechanism of penetration; (iii) they conduct a controlled analysis of the physical forces involved in penetration and report that they are in the low nM range, not especially strong but much higher that the mosquito bite and finally they provide a first analysis of blood uptake during feeding.

Strengths:

The study images the tsetse fly feeding structures in unprecedented detail, with resolution to the uM scale, in 3-D, and during feeding. The resulting images are dramatic and insightful (and beautiful and frightening!), so researchers interested in trypanosomes, tsetse flies, or blood feeding by flies in general will want to see.

They conclude that flies attach strongly to smooth surfaces because of interactions possible via the array of acanthae of the pulvillus pad at the ends of the tarsi. The estimated attachment forces are similar in male & female flies, in the low mM range (they look impressively strong in video 1). They provide a very striking analysis of the proboscis and labellum and associated tooth structures (Figures 4 & 5). I recall many years ago observing that tsetse flies are messy feeders, and these structures, especially the rasping teeth structures on the reverse folded labial tips, explain why! This seems more like a chainsaw than a jigsaw in action, but the authors are probably correct that these structures and the probing/retraction mechanism explain many features of tsetse fly feeding and their ability to feed on a wide range of hosts with very different skin types.

We agree that “jigsaw” may be too specific and not fully appropriate in this context. We have therefore replaced it in the manuscript with the more general term “saw.”

The impressive aspect of this paper is the range of imaging techniques (CLSM, SEM, uCT, FIB SEM), the quality of the images, which attests to the obvious care taken with sample preparation. The biomechanical analysis, especially the penetration analysis, is impressive. Finally, the paper is clearly written and presented; it was a very easy read and, overall, a very engaging study.

Weaknesses:

I suppose it could be said that the paper is a descriptive study; it doesn't really test a hypothesis, but that is not a prerequisite for sharing it. Perhaps the least convincing parts are the imaging of the flexible versus rigid parts of the structures, which is based on the amount of resilin (flexible) and chitin-protein (stiff), based on their autofluorescence. It seems odd that the joints would be less blue (stiffer) in Figure 1i, or what the blue structures correspond to in Figure 6B-D.

Our analysis is based on established CLSM approaches that use exoskeleton autofluorescence as a proxy for relative differences in cuticular composition and material properties (Michels & Gorb, 2012; Michels et al., 2016). In the tarsus, the observed differences in inferred stiffness are relatively subtle, with most regions exhibiting broadly comparable material properties. This becomes particularly evident when compared with the proboscis, where the contrasts in cuticular composition are much more pronounced (Figure 6). We also note that locally stiffer regions at joints are not unexpected, as stiffness gradients in arthropod joints can provide mechanical support and help constrain the direction of movement. Importantly, our images show a flexible, ring-like blue region directly at the articulation, surrounded by slightly stiffer material. We therefore interpret this pattern as a combination of a flexible hinge region and adjacent supporting structures that together enable controlled joint motion.

The blue structures in Figure 6B–D correspond to flexible regions of the furca (f). Because this spring-like cuticular element undergoes substantial configuration changes during labellar eversion, the presence of highly flexible regions is consistent with its proposed mechanical function.

**Recommendations for the authors:**

**Reviewer #1 (Recommendations for the authors):**

No further experiments or analyses are suggested. However, the Discussion would benefit from briefly acknowledging how trypanosome infection can alter feeding behavior and mouthpart function, based on prior work, to place the mechanical findings in a more biologically relevant transmission context.

We thank the reviewer for this suggestion. Previous studies have indeed shown that trypanosome infection can alter tsetse feeding behavior, primarily through changes in saliva composition. Van den Abbeele et al. (2010) demonstrated that infection with *T. brucei* significantly impairs the anti-haemostatic activity of tsetse saliva, resulting in prolonged prefeeding probing and therefore extended feeding times. These findings are consistent with earlier observations by Jenni (1980), who reported increased probing frequency in infected flies.

Jenni (1980) also proposed that these behavioral changes might be linked to altered mechanoreceptor function. However, Van den Abbeele et al. (2010) argued against this interpretation for *T. brucei*, noting that this parasite does not colonize the mouthparts where these mechanoreceptors are located. Taken together, the available evidence suggests that the observed changes in feeding behavior are mediated primarily through altered interactions with host blood rather than through direct effects on the mouthparts themselves.

It should be noted that this conclusion is specific to *T. brucei*. Other tsetse-transmitted trypanosome species, such as *T. congolense*, do colonize the proboscis. However, a comparative study examining flies infected with *T. brucei*, *T. congolense*, or *T. vivax* found no significant effects of infection on feeding behaviour relative to uninfected controls (Moloo, 1983). To our knowledge, there is currently also no direct evidence that any trypanosome species alters the physical properties or mechanical function of the mouthparts, or causes damage that would directly affect feeding performance. We have added this paragraph to the Discussion (lines 508520):

"It is also important to note that our experiments were conducted using uninfected flies. Previous studies have shown that trypanosome infection can alter feeding behaviour, including increased probing activity and prolonged feeding times (Jenni et al., 1980; Van den Abbeele et al., 2010). These effects have primarily been attributed to infection-induced changes in saliva composition and the resulting interactions with host blood (Van den Abbeele et al., 2010). However, a different study found no significant effects of infection with either salivary gland-resident *T. brucei* or with proboscis-colonizing species such as *T. congolense* and *T. vivax* on Glossina feeding behaviour (Moloo, 1983). Furthermore, although infection-associated transcriptional changes in the salivary glands and proboscis have been reported (Awuoche et al., 2017; Matetovici et al., 2016), there is currently no direct evidence that trypanosome infection alters the mechanical properties or function of the mouthparts themselves."

**Reviewer #2 (Recommendations for the authors):**

Several figures (particularly SEM-based ones) contain very dense labeling. Consider providing simplified overviews or annotated "orientation guides" in figure supplements to improve navigability for readers unfamiliar with proboscis anatomy.

We thank the reviewer for this helpful suggestion. While we agree that orientation aids can be valuable, we have decided not to include additional simplified overview figures, as we consider that introducing separate schematic summaries could potentially complicate rather than improve navigation of the structural detail. We therefore rely on consistent labelling within the existing figures and detailed captions to guide interpretation.

The manuscript uses appropriate non-parametric tests, but could benefit from reporting effect sizes and indicating sample sizes on all plots.

Sample sizes are reported in the figure legends, Methods section, and Supplementary material for all experiments. We agree that reporting effect sizes can be informative and will consider this in future studies. However, because the primary objective of the statistical analyses in the present work was to support comparisons between experimental conditions rather than to estimate effect magnitudes, and because the figures are already information-dense, we therefore decided not to further modify the graphical presentation in this revision.

**Reviewer #3 (Recommendations for the authors):**

(1) P5 L111. Perhaps indicate these knobs on the image Figure 1S. I assume these are the structures visible under the pointer labelled spa? Maybe highlight some of the worn areas in Figure 1G.

The knob-like structures in Figure S1 are highlighted in green and we have now revised the figure description from:

“…showing fine crests on the underside and surface modifications (green) on the upper side.”

to:

“…showing fine crests on the underside and knob-like surface modifications (green) on the upper side.”

Regarding Figure 1G, the purpose of the panel is to illustrate the contrast between deformed spatulae (Figure 1G) and intact spatulae (Figure 1H). We therefore chose to retain the original presentation, as we feel that additional markings would not substantially improve interpretation and could obscure structural details. We hope that the direct comparison between the two panels provides sufficient visual guidance.

(2) P9. The frictional force (and P38/39) has the units of N (kg.m/Sexp2). The safety factor is this force divided by the weight of the fly? So are there units (Kg/sexp2) or are these not shown? Perhaps this is a convention.

The safety factor is defined as the ratio of the total frictional force to the fly’s weight force (m·g), where m is body mass and g is gravitational acceleration. Since both quantities are express in Newtons (kg·m·s^-2^), the safety factor is dimensionless.

We agree that the terminology in the original manuscript may have been ambiguous, as “body weight” is sometimes used colloquially to refer to body mass. To avoid confusion, we have revised the text to explicitly refer to weight force and now define the safety factor as the total friction force divided by weight force (mg, where m is body mass and g is gravitational acceleration). We have clarified this in the main text, the Figure 2 legend, and the description of Supplementary Material 1.

(3) P10 Figure 2G & H. It is not very clear...are these the data, the average of all readings across all surfaces in E and F? If so, why is this value useful...how does it add to what is already shown?

The figures 2G and 2H summarize the friction forces (G) and safety factors (H) across all tested substrates, based on the values from the male (B, E) and female (C, F) datasets. The purpose of these panels is to provide an overall comparison between sexes independent of substrate type. While this information can also be inferred from the substrate-specific plots, the sex-separated presentation does not make the absence of an overall sex difference immediately obvious. Figures 2G and 2H therefore serve as concise summary plots highlighting this result.

(4) P12. For the nonspecialist, it might be useful to draw a cartoon showing the organisation of the labium, labrum and the hypopharynx...this is visible in Figure 4i but not in the dissected proboscis and labellum ....only the labium as the labrum doesn't extend this far?

To clarify the anatomical arrangement in the dissected specimen, we have added the following statement to the Figure 4 legend (lines 224–226):

“In an intact fly, the labrum would be positioned within the empty groove of the labium visible in J; however, it is absent in this dissected preparation.”

(5) P17 legend to Figure 5. Include the Lm abbreviation in the legend, and maybe a close-up of the rsp teeth?

We have added “lm, labellum” to the Figure 5 legend (line 250), as this abbreviation was previously missing. Panel J is a close-up of the rasping teeth.

(6) F3S and Video 3. Are the images in B and C taken from the FIB SEM video images? It is not clear. A small legend descriptor for video 3 would be helpful.

The images in Supplementary Figure 3B and C are reconstructed from the same FIB-SEM dataset shown in Video 3, but they are displayed in a different orientation. This is indicated schematically in Supplementary Figure 3A, which illustrates the viewing plane used for the reconstruction.

We already included the following legend for Video 3 (lines 1146–1149):

"Video 3: FIB-SEM of the tsetse labellum. Sequential cross sections reveal internal ultrastructure progressing from near the tip of the labellum downward. Data were acquired on a Crossbeam 540 (Zeiss) with the EsB detector in continuous milling mode."

To improve clarity, we have now added a sentence to the video legend linking the figures to the video: (lines 1149-1150)

“Reconstructed images from this dataset are shown in Figure 5A and Supplementary Figure 3B and C.”

In addition, we have now explicitly cross-referenced Video 3 in the legends of Figures 5 and S3 to make the connection clearer for the reader.

(7) Figure 7. These are amazing images, especially G-I.

Thank you for this positive feedback, we appreciate it.

(8) P24. It is really good to see that there is a difference in force penetration for full skin v dermal...this deserves a comment.

We agree and have revised the text accordingly. We replaced:

"Human skin substrates required the lowest penetration forces, with 0.97 mN for full-thickness skin equivalents, 0.67 mN for dermal equivalents, and 0.85 mN for native skin explants (Figure 8C, D)."

With this (lines 363-367):

"Human skin substrates showed the lowest penetration forces, with dermal equivalents requiring less force (0.67 mN) than full-thickness skin equivalents (0.97 mN), reflecting the additional mechanical resistance of the epidermal layer absent in dermal-only constructs. Native skin explants fell intermediate at 0.85 mN (Figure 8C, D)."

(9) P26 Figure S5. Panel c, there seems to be a big scatter in the drinking time. Was there an outlier?

Indeed, the observed scatter is due to a single fly with an unusually long drinking time of 184.44 seconds, which is approximately six times the median duration. We have verified the underlying data and found no indication of a measurement error; the value therefore remains included in the analysis. The data for the plots in Supplementary Figure 5 are also available in Supplementary Material 3.
